# Viral Load Levels Measured at Set-Point Have Risen Over the Last Decade of the HIV Epidemic in the Netherlands

**DOI:** 10.1371/journal.pone.0007365

**Published:** 2009-10-07

**Authors:** Luuk Gras, Suzanne Jurriaans, Margreet Bakker, Ard van Sighem, Daniela Bezemer, Christophe Fraser, Joep Lange, Jan M. Prins, Ben Berkhout, Frank de Wolf, for the ATHENA national observational cohort study

**Affiliations:** 1 Stichting HIV Monitoring, Amsterdam, the Netherlands; 2 Department of Medical Microbiology, Centre for Infection and Immunity Amsterdam (CINIMA), Academic Medical Centre of the University of Amsterdam, Amsterdam, the Netherlands; 3 Department of Infectious Diseases Epidemiology, Imperial College School of Medicine, London, United Kingdom; 4 Department of Internal Medicine, Division of Infectious Diseases, Tropical Medicine and AIDS, and Centre for Infection and Immunity Amsterdam (CINIMA), Academic Medical Centre, Amsterdam, the Netherlands; McGill University Health Center, Montreal Chest Institute, Canada

## Abstract

**Background:**

HIV-1 RNA plasma concentration at viral set-point is associated not only with disease outcome but also with the transmission dynamics of HIV-1. We investigated whether plasma HIV-1 RNA concentration and CD4 cell count at viral set-point have changed over time in the HIV epidemic in the Netherlands.

**Methodology/Principal Findings:**

We selected 906 therapy-naïve patients with at least one plasma HIV-1 RNA concentration measured 9 to 27 months after estimated seroconversion. Changes in HIV-1 RNA and CD4 cell count at viral set-point over time were analysed using linear regression models. The ATHENA national observational cohort contributed all patients who seroconverted in or after 1996; the Amsterdam Cohort Studies (ACS) contributed seroconverters before 1996. The mean of the first HIV-1 RNA concentration measured 9–27 months after seroconversion was 4.30 log_10_ copies/ml (95% CI 4.17–4.42) for seroconverters from 1984 through 1995 (n = 163); 4.27 (4.16–4.37) for seroconverters 1996–2002 (n = 232), and 4.59 (4.52–4.66) for seroconverters 2003–2007 (n = 511). Compared to patients seroconverting between 2003–2007, the adjusted mean HIV-1 RNA concentration at set-point was 0.28 log_10_ copies/ml (95% CI 0.16–0.40; p<0.0001) and 0.26 (0.11–0.41; p = 0.0006) lower for those seroconverting between 1996–2002 and 1984–1995, respectively. Results were robust regardless of type of HIV-1 RNA assay, HIV-1 subtype, and interval between measurement and seroconversion. CD4 cell count at viral set-point declined over calendar time at approximately 5 cells/mm^3^/year.

**Conclusion:**

The HIV-1 RNA plasma concentration at viral set-point has increased over the last decade of the HIV epidemic in the Netherlands. This is accompanied by a decreasing CD4 cell count over the period 1984–2007 and may have implications for both the course of the HIV infection and the epidemic.

## Introduction

During the asymptomatic phase of HIV-1 infection, virus production and clearance are believed to reach a balance reflecting a relatively stable level of HIV-1 RNA concentration in plasma. Whether this balance, or viral set-point, is reached in all patients remains open to debate [1], [2]. It is agreed, however, that with a higher HIV-1 RNA plasma level, progression to AIDS is more frequent [3], as is the rate of HIV-1 transmission [4]. A rising trend over time in plasma HIV-1 RNA concentration at set-point might imply an increase in the efficiency of transmission [5], [6]. Three observational studies found no evidence for such a change [7]–[9], whereas two studies did [10], [11]. Contrasting results likewise come from studies of HIV-1 RNA replicative fitness at viral set-point, thought to be positively correlated with HIV-1 RNA concentration in plasma [12], [13]. One study suggested a lower replicative fitness in HIV-1 isolates obtained from patients infected in 2002–2003 compared to isolates from patients infected between 1986–1999 [14], but samples were not matched for time since seroconversion. A similar study, using isolates obtained from participants of the Amsterdam Cohort Study and samples matched for time since seroconversion, found an increase in replicative fitness over time [15].

Here we present a study of changes in the mean HIV-1 RNA concentration and CD4 cell count at viral set-point measured in patients who became seropositive between 1984 and 2007.

## Results

Baseline characteristics of the included 906 patients are summarized in Table 1. CD4 cell counts were available for 811 (90%). Of the 906 total, 92% were male, 76% had homosexual contact recorded as the most likely transmission route, and 82% originated from W-Europe/N-America. Most patients from other regions of origin were from S-America/Caribbean. Only 2% were from sub-Sahara Africa.

**Table 1:** Baseline characteristics.

	Estimated 1984–1995	year of 1996–2002	seroconversion 2003–2007	Total
**Total**	163	232	511	906
**MSM from W-Europe/N-America, excluding non-B subtype**	114 (71%)	143 (61%)	355 (66%)	612 (68%)
**Gender**				
Male	144 (88%)	206 (89%)	480 (94%)	830 (92%)
**Transmission risk group**				
MSM	119 (73%)	162 (70%)	410 (80%)	691 (76%)
Heterosexual	3 (2%)	49 (21%)	54 (11%)	106 (12%)
IDU	22 (13%)	7 (3%)	2 (0%)	31 (3%)
Other	17 (11%)	12 (5%)	18 (4%)	47 (5%)
Unknown	2 (1%)	2 (1%)	27 (5%)	31 (3%)
**Region of origin**				
W-Europe/N-America	134 (82%)	188 (81%)	420 (82%)	742 (82%)
Other	5 (3%)	40 (17%)	75 (15%)	120 (13%)
Unknown	24 (15%)	4 (2%)	16 (3%)	44 (5%)
**Subtype**				
B	59 (36%)	76 (33%)	273 (53%)	408 (45%)
Non-B	1 (1%)	8 (3%)	32 (7%)	41 (5%)
Sample not available	103 (63%)	148 (64%)	206 (40%)	457 (50%)
**Resistance-associated mutation found**				
At least one mutation	7 (4%)	5 (2%)	20 (4%)	32 (4%)
None	46 (28%)	78 (34%)	269 (53%)	393 (43%)
Sequence not available	110 (67%)	149 (64%)	222 (43%)	481 (53%)
**Sensitivity of assay**				
Standard	163 (100%)	104 (45%)	42 (8%)	309 (34%)
Sensitive	0	114 (49%)	443 (87%)	414 (46%)
Unknown	0	14 (6%)	26 (5%)	40 (4%)
**Amplification technique of assay**				
NASBA	163 (100%)	53 (23%)	44 (9%)	260 (29%)
bDNA	0	66 (28%)	175 (34%)	241 (27%)
RT-PCR	0	99 (43%)	266 (5%)	265 (29%)
Unknown	0	14 (6%)	26 (5%)	40 (4%)
**HBV**				
Negative	59 (36%)	201 (87%)	431 (84%)	691 (76%)
Positive	3 (2%)	14 (6%)	24 (5%)	41 (5%)
Unknown	101 (62%)	17 (7%)	66 (11%)	184 (20%)
**HCV**				
Negative	44 (27%)	188 (81%)	397 (78%)	629 (69%)
Positive	9 (6)	10 (4%)	20 (4%)	39 (4%)
Unknown	110 (67)	34 (15%)	94 (18%)	238 (26%)
**Age at seroconversion in years median, (IQR)**	34.4 (28.9–40.5)	33.8 (29.9–40.4)	36.4 (30.0–43.1)	35.2 (29.8–41.7)
**Months between seroconversion and plasma HIV-1 RNA measurement, median (IQR)**	11.6 (10.1–14.5)	10.9 (9.9–12.7)	10.9 (9.8–12.4)	10.9 (9.9–12.8)
**Months between seroconversion and CD4 cell count measurement, median (IQR)**	10.3 (10.0–11.1)	10.7 (9.8–12.2)	10.6 (9.7–11.9)	10.5 (9.8–11.8)

Results of HIV-1 subtyping, using nucleotide sequences of the *pol* region obtained for HIV-1 drug-resistance testing, were available for 449 (50%) patients, and subtype B was found in 408 (91%). Infection with circulating recombinant form (CRF) 02_AG was found in 15 patients, CRF 01_AE in 8, subtype A in 5, subtype C in 5, subtype G in 4, subtype D in 2, subtype A1 in 1 and CRF 03_AB in 1 patient. Of 425 patients tested before antiretroviral therapy was started, 32 (7.5%) had at least one resistance mutation.

In all 163 patients with seroconversion before 1996, the HIV-1 RNA concentration at set-point was measured with assays using the NASBA technique. Overall, RT-PCR was most used (in 40% of the 906 total). HIV-1 RNA plasma concentrations measured at set-point were below the lower quantitation limit of the assay used in 37 of 906 (4%) patients. In 19 patients (2%), HIV-1 RNA concentrations were above the upper quantitation limit of the assay used.

The mean HIV-1 RNA concentration at set-point in all 906 patients was 4.45 log_10_ copies/ml. It was 4.30, 4.27, and 4.59 log_10_ copies/ml in patients with an estimated seroconversion date between 1984–1995, 1996–2002, and 2003–2007, respectively. Table 2 shows the differences in mean HIV-1 RNA concentration according to estimated year of seroconversion, as obtained with unadjusted and adjusted regression models. Compared to patients with an estimated seroconversion date in or after 2003, the adjusted mean HIV-1 RNA concentration among patients seroconverting between 1996 and 2002 and before 1996 was lower by 0.29 log_10_ copies/ml (95% CI 0.16–0.41; p<0.0001) and 0.27 (0.12–0.42; p = 0.0004), respectively. Furthermore, the adjusted mean HIV-1 RNA concentration at set-point was 0.32 (95% CI 0.12–0.51) log_10_ copies/ml lower in women compared to men (p = 0.002). Patients infected with subtype B had on average a 0.40 (0.14–0.67) log_10_ copies/ml higher HIV-1 RNA concentration (p = 0.003) than patients infected with non-B subtypes. The mean HIV-1 RNA concentration was 0.16 (0.00–0.32) log_10_ copies/ml higher in patients from W-Europe/N-America compared to patients from elsewhere (p = 0.04). There were no significant differences in mean HIV-1 RNA concentration according to age at seroconversion (p = 0.43), HIV transmission group (p = 0.95), interval between seroconversion and viral set-point (p = 0.96), or presence of a resistance mutation (p = 0.92).

**Table 2:** Mean (95% CI) differences in HIV-1 RNA concentration at viral set-point (log_10_ copies/ml) according to time of seroconversion.

	Plasma HIV-1 RNA concentration	First 9–27 months	after seroconversion	at 12 months	at 18 months	at 24 months
	Patient group	All patients	Homogeneous patient group^a^	Homogeneous patient group^a^	Homogeneous patient group^a^	Homogeneous patient group^a^
	**N**	906	612^b^	552^c^	370^c^	315^c^
**Unadjusted model**	**Year of seroconversion**					
	2003–2007 (reference)	0.00	0.00	0.00	0.00	0.00
	1996–2002	−0.32 (−0.45, −0.20)	−0.44 (−0.59, −0.29)	−0.45 (−0.61, −0.29)	−0.35 (−0.54, −0.15)	−0.39 (−0.62, −0.16)
		p<0.0001	p<0.0001	p<0.0001	p = 0.0005	p = 0.0008
	1984–1995	−0.29 (−0.44, −0.15)	−0.37 (−0.54, −0.20)	−0.37 (−0.54, −0.20)	−0.38 (−0.61, −0.15)	−0.32 (−0.54, −0.10)
		p<0.0001	p<0.0001	p<0.0001	p = 0.001	p = 0.008
**Adjusted model**	**Year of seroconversion**					
	2003–2007 (reference)	0.00	0.00	0.00	0.00	0.00
	1996–2002	−0.29 (−0.41, −0.16)	−0.41(−0.67, −0.27)	−0.42 (−0.58, −0.26)	−0.33 (−0.53, −0.13)	−0.39 (−0.62, −0.16)
		p<0.0001	p<0.0001	p<0.0001	p = 0.001	p = 0.0008
	1984–1995	−0.27 (−0.42, −0.12)	−0.34 (−0.51, −0.17)	−0.34 (−0.51, −0.17)	−0.37 (−0.60, −0.13)	−0.32 (−0.54, −0.10)
		p = 0.0004	p<0.0001	p<0.0001	p = 0.002	p = 0.005
**Adjusted model also including type of assay**	**Year of seroconversion**					
	2003–2007 (reference)	0.00	0.00	0.00	0.00	0.00
	1996–2002	−0.31 (−0.44, −0.18)	−0.44(−0.60, −0.28)	−0.46 (−0.62, −0.29)	−0.34 (−0.54, −0.13)	−0.37 (−0.61, −0.13)
		p<0.0001	p<0.0001	p<0.0001	p = 0.001	p = 0.0008
	1984–1995	−0.40 (−0.63, −0.18)	−0.55 (−0.82, −0.29)	−0.60 (−0.87, −0.32)	−0.40 (−0.76, 0.03)	−0.32 (−0.74, 0.11)
		p = 0.0003	p<0.0001	p<0.0001	p = 0.002	p = 0.005

a Homogeneous patient group: MSM from W-Europe/N-America. Patients with non-B subtype infection excluded.

b Adjusted for gender, region of origin, subtype, age at seroconversion, HIV transmission group, interval between seroconversion and viral set-point, and presence of a resistance mutation.

c Adjusted for availability of subtype data.

To test whether the increase in viral set-point could reflect changing use of various quantitative HIV-1 RNA assays over time, we added type of assay to the model. The differences in mean HIV-1 RNA concentration between different periods of seroconversion increased slightly (Table 2). Relative to seroconverters between 2003 and 2007, the mean HIV-1 RNA concentration was 0.31 log_10_ copies/ml (95% CI 0.18–0.44; p<0.0001) lower for seroconverters between 1996 and 2002 and 0.40 (0.18–0.63; p = 0.0003) lower for those seroconverting before 1996. The difference in HIV-1 RNA concentration measured with RT-PCR assays was on average −0.12 log_10_ copies/ml (95% CI −0.30, 0.07; p = 0.21) compared to NASBA assays and 0.04 (−0.09, 0.17; p = 0.54) compared to bDNA assays. The HIV-1 RNA concentration was on average 0.16 log_10_ copies/ml (−0.03, 0.35; p = 0.10) higher when measured with the NASBA technique compared to samples tested with assays using bDNA. HIV-1 RNA concentration measured using assays with a lower detection limit ≤400 copies/ml was 0.08 log_10_ copies (95% CI −0.24, 0.08; p = 0.33) lower than those measured using assays with a higher detection limit. However, the differences according to calendar year of seroconversion remained similar. Also, when analyses were stratified according to the type or sensitivity of the assay, results remained similar (results not shown).

Given scarce data on HCV or HBV co-infection in patients who seroconverted before 1996, we restricted analyses including such co-infections as a confounder to the 743 patients who seroconverted in or after 1996. Mean HIV-1 RNA concentration in patients with a HCV co-infection was 0.36 log_10_ copies/ml (95% CI 0.08–0.64, p = 0.01) higher than in patients without a HCV co-infection. It was 0.09 log_10_ copies/ml (−0.16−0.35, p = 0.46) higher in patients with a HBV co-infection compared to patients without. Differences in mean HIV-1 RNA concentration according to year of HIV-1 seroconversion remained similar, being 0.29 log_10_ copies/ml (0.17–0.41, p<0.0001) higher in patients with seroconversion between 2003–2007 compared to 1996–2002.

**Figure 1:**
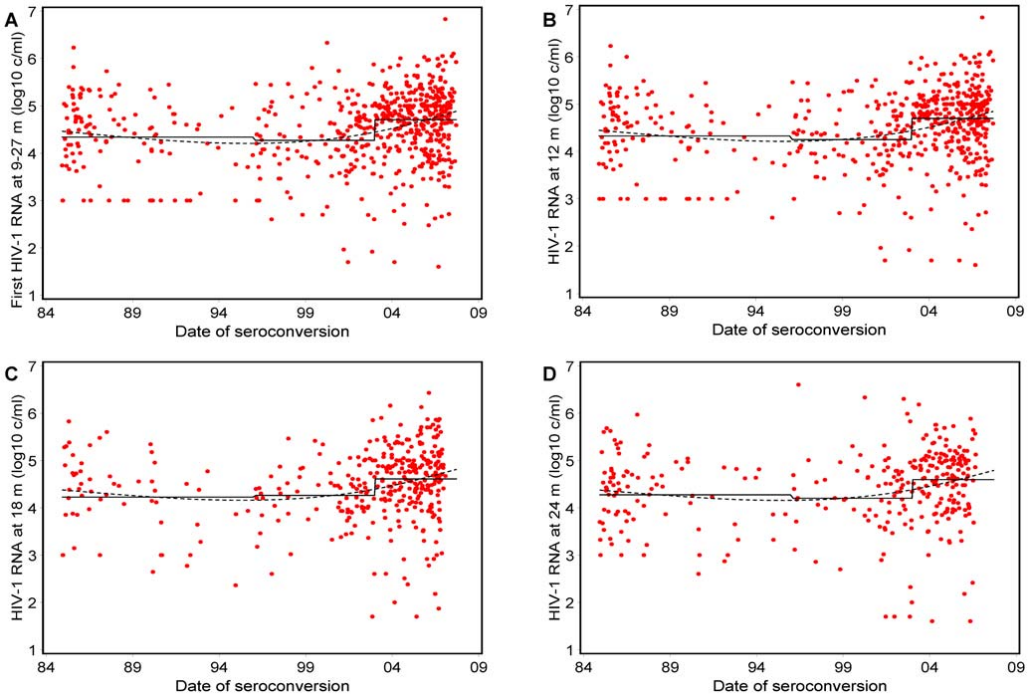
HIV-1 RNA concentration at viral set-point and mean HIV-1 RNA concentration at each time period. In MSM patients from W-Europe or N-America with a proven or likely infection with subtype B: a) first HIV-1 RNA 9–27 months after seroconversion (n = 612), b) at 12 (n = 552), c) 18 (n = 370), and d) 24 months (n = 315). The solid black line shows the mean HIV-1 RNA concentration for patients with an estimated date of seroconversion from 1984 through 1995, 1996 through 2002, and 2003 through 2007 (as shown in Table 2). Dashed black lines are estimates obtained by continuous modelling of the estimated date of seroconversion using cubic splines.

To avoid any bias arising from changes in the distribution of ethnicity, gender, and infection with non-B subtype over time, we focused on a homogeneous patient group of 612 MSM from W-Europe/N-America. Patients with a confirmed HIV-1 non-B infection were excluded. Figure 1 shows the HIV-1 RNA concentration at set-point and at 12, 18, and 24 months after seroconversion and the estimated mean HIV-1 RNA concentration by calendar year of seroconversion. The dashed line in Figure 1a shows that the mean set-point HIV-1 RNA concentration at the start of 1985 was 4.46 log_10_ copies/ml (95% CI 4.27–4.65). It was 4.21 log_10_ copies/ml (4.09–4.33) at its lowest value in 1995 and 4.88 log_10_ copies/ml (4.76–5.01) in 2007. Estimates of differences in mean HIV-1 RNA concentration at set-point according to the calendar year of seroconversion for this subgroup, shown in Table 2, are similar to those for the total of 906 patients. HIV-1 subtype was the only variable included in the adjusted model apart from estimated year of seroconversion. On further restricting the sample size to the 297 patients known to be infected with subtype B, we found similar differences in mean HIV-1 RNA concentration (results not shown). Finally, we looked separately at plasma HIV-1 RNA levels at 12, 18, and 24 months after seroconversion and found mean HIV-1 RNA concentrations of 4.50, 4.48, and 4.40 log_10_ copies/ml, respectively. Differences in mean HIV-1 RNA concentration at 12 and 18 months according to estimated year of seroconversion were similar to those obtained through models including the first HIV-1 RNA concentration between 9 and 27 months after seroconversion. In a final sensitivity analysis, including 751 patients with a maximum seroconversion interval of 6 months, the mean of the first HIV-1 RNA concentration taken after seroconversion was 0.48 log_10_ copies/ml (95% CI 0.26–0.71; p<0.0001) lower for seroconverters before 1996 and 0.17 (0.00–0.35; p = 0.05) lower between 1996–2002 compared to 2003–2007. The mean was 0.31 log_10_ copies/ml (0.06–0.56; p = 0.02) lower for seroconverters before 1996 compared to 1996–2002.

**Figure 2:**
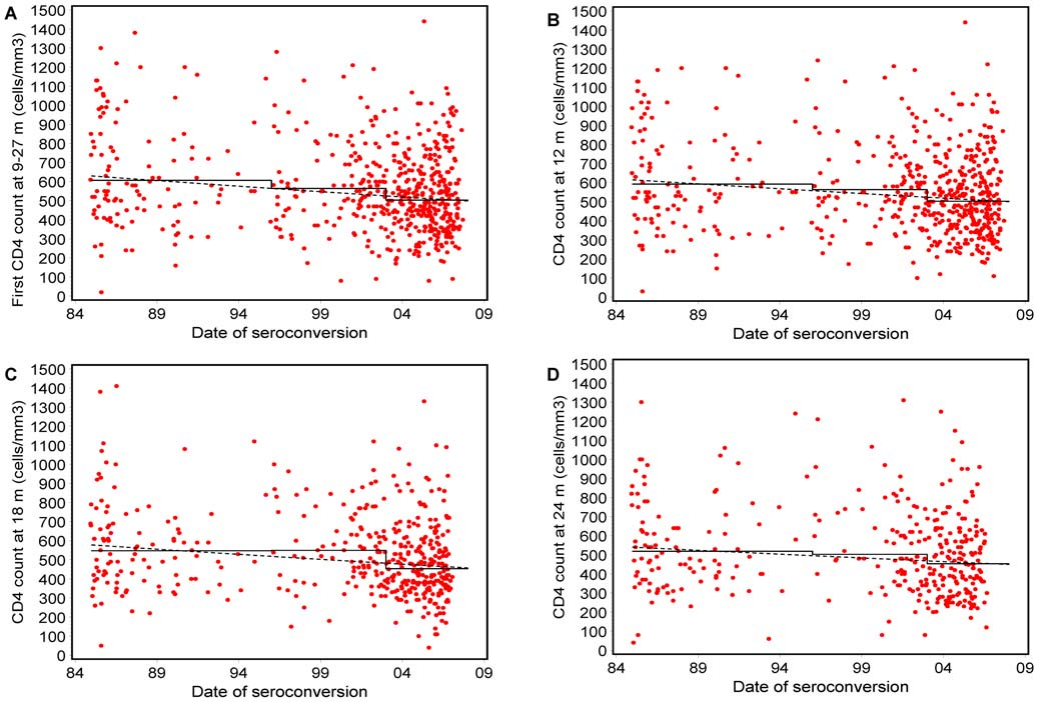
CD4 cell count at viral set-point and mean CD4 cell count at each time period. In MSM from W-Europe/N-America with a proven or likely subtype B infection: a) first CD4 cell count between 9 and 27 months after seroconversion (n = 578), b) at 12 months (n = 555), c) 18 months (n = 439), and d) 24 months (n = 347). The solid black line shows the mean CD4 cell count for patients with an estimated date of seroconversion from 1984 through 1995, 1996 through 2002, and 2003 through 2007 (as shown in Table 2). The dashed black lines were obtained using linear models assuming a constant decrease between 1984 and 2007.

In 811 patients with CD4 cell counts available between 9–27 months after seroconversion, the median count at viral set-point was 520 cells/mm^3^ (IQR 390–680). Table 3 shows results of the linear regressions of CD4 cell count at viral set-point. Mean CD4 cell count at viral set-point declined throughout the period 1984–2007 by 0.025 cube root cells/mm^3^/year (95% CI 0.012, 0.038; p<0.0001), a decline of approximately 5 CD4 cells/mm^3^/year. Region of origin was the only other variable included in the adjusted model. Mean CD4 cell count at viral set-point in patients from W-Europe/N-America with seroconversion between 2003–2007 was 507 cells/mm^3^ (485–530), compared to 466 cells/mm^3^ (425–509, difference p = 0.07) for patients from elsewhere. Table 3 shows results of regression analyses of CD4 cell count on a cube-root scale for our homogeneous patient group. The mean decrease over time of the first CD4 cell count 9–27 months after seroconversion and at 12, 18, and 24 months after seroconversion was 0.028 (95% CI 0.014, 0.041), 0.025 (0.011, 0.038), 0.027 (0.013, 0.041), and 0.021 (0.004, 0.038) cubic root cells/mm^3^/year, respectively. Figure 2 shows the estimates back-transformed to the original scale. Mean CD4 count at 12 months was 592 (562–653), 563 (523–605) and 502 cells/mm^3^ (479–527) for seroconverters between 1984–1995, 1996–2002, and 2003–2007, respectively. Estimated differences in CD4 count between seroconverters of 1996–2002 and 2003–2007 were greater in analyses of the homogeneous patients than in analyses of the total group.

**Table 3:** Changes (95% CI) in CD4 cell count at viral set-point (cells/mm^3^) using different models.

CD4 cell count	First	9–27 months after	seroconversion	at 12 months	at 18 months	at 24 months
Patient group	All patients	All patients	Homogeneous patient group^a^	Homogeneous patient group^a^	Homogeneous patient group^a^	Homogeneous patient group^a^
	Unadjusted	Adjusted^b^				
**N**	811	811	578	555	439	347
**Median CD4 cell count, (cells/mm^3^)**	520	520	530	530	490	480
**Change in CD4 cell count at viral set-point (cubic cells/mm^3^/year)**	−0.025 (−0.038, −0.012)	−0.026 (−0.039, −0.013)	−0.028 (−0.041, −0.014)	−0.025 (−0.038, −0.011)	−0.027 (−0.041, −0.013)	−0.021 (−0.038, −0.004)
	p<0.0001	p = 0.0001	p<0.0001	p = 0.0004	p = 0.0002	p = 0.02
**Difference in mean CD4 cell count (cube root cells/mm^3^)**						
**2003**–**2007 (reference)**	0.00	0.00	0.00	0.00	0.00	0.00
**1996**–**2002**	0.18 (−0.01, 0.37)	0.19 (−0.01, 0.38)	0.31 (0.08, 0.54)	0.31 (0.07, 0.54)	0.50 (0.25, 0.76)	0.27 (−0.05, 0.59)
	p = 0.07	p = 0.06	p = 0.008	p = 0.01	p<0.0001	p = 0.10
**1984**–**1996**	0.43 (0.20, 0.65)	0.45 (0.21, 0.68)	0.51 (0.26, 0.76)	0.45 (0.20, 0.70)	0.49 (0.24, 0.74)	0.35 (0.05–0.65)
	p = 0.0002	p = 0.0002	p<0.0001	p = 0.0004	p = 0.0001	p = 0.02

a Homogeneous patient group: MSM from W-Europe/N-America. Patients with non-B subtype infection excluded.

b Adjusted for gender, region of origin, subtype, age at seroconversion, HIV transmission group, interval between seroconversion and viral set-point, and presence of a resistance mutation.

## Discussion

We found a rising trend over time in the HIV-1 RNA concentration at set-point in patients infected in the last decade, with a complementary downward trend in CD4 cell count at viral set-point.

Our results agree with those of the CASCADE study [10] and a recent study of the epidemic in Italy [11]. The CASCADE study found an increase in mean HIV-1 RNA concentration at viral set-point of 0.035 log_10_ copies/ml/year over the period 1985–2002, although we found an increase only from 1996. Three other studies found no evidence for an increase [7]–[9]. Differences in patient selection, study period, and outcome definitions across these five studies might explain the discrepancies. In a study from the Swiss HIV Cohort Study (SHCS) and the Italian cohort study [8], [11], all patients with a confirmed HIV-1 infection were selected. Other studies restricted patient selection to seroconverters with a maximum seroconversion interval of 6 months [9] or 12 months [7], [10]. Herbeck *et al*. [7] looked at HIV-1 RNA concentration at set-point in 384 homosexual patients with known seroconversion dates between 1985 and 2005, but most were infected before 1996. The study period in that study and the SHCS [8] might have been too short to find an increase in HIV-1 RNA concentration at set-point over time. Outcome definitions of the five studies ranged from the first available measurement of HIV-1 RNA plasma concentration after seroconversion [9], [10] to measurements at a later stage [7], [8], [11]. Because the exact moment of seroconversion is unknown, the former definition has the disadvantage of not knowing whether the measurement was taken during the peak HIV RNA concentration phase following infection, during the phase shortly before or after the peak, or during the set-point phase. This type of measurement error most likely hampers the detection of significant changes over time. Admittedly, using measurements at a later stage can introduce bias, because patients who have started antiretroviral therapy early are censored from the analysis. Assuming that patients with a high HIV-1 RNA concentration and a low CD4 cell count will start therapy earlier than patients with a lower concentration and a higher cell count, results may be biased towards a lower HIV-1 RNA concentration and higher CD4 cell count at viral set-point from 1996 onwards, especially in measurements taken 24 months after seroconversion. This may explain our finding of a significant difference between patients who seroconverted before 1996 and those who did so between 1996 and 2002 with respect to the first HIV-1 RNA concentration after seroconversion and no significant difference when analysing the first HIV-1 RNA concentration taken 9–27 months after seroconversion. However, both analyses showed an increasing HIV-1 RNA concentration over time.

The decreasing trend in CD4 cell count at viral set-point over time complements the increasing trend in HIV-1 RNA concentration. A similar decrease in CD4 cell count over time was likewise found in other studies [10], [16], [17]. Evidence of an increasing trend [18] or a stable level [7], [19] in CD4 cell count might reflect a shorter study period.

Bias through systematic inclusion of correlated transmission networks is unlikely, as active enrolment of related partners was never in place during our entire study period. Other potential sources of bias which could have influenced our finding include the genetic heterogeneity of HIV-1, which can impede the accuracy of quantitation, especially in early assays primarily designed to detect subtype B [20]–[22]. However, the effect persisted when we focused on a homogeneous group of MSM from W-Europe/N-America with a proven or highly likely subtype B infection. The higher mean HIV-1 RNA concentration in patients for whom we lacked subtype data points to some residual confounding because of infection with non-B subtypes. However, sensitivity analyses of patients known to be infected with subtype B found similar differences in mean HIV-1 RNA concentration at set-point over time.

Disease progression has been shown to differ among patients with subtype A, C, D and G infection [23]–[25]. We found a higher mean HIV RNA concentration in patients with subtype B infection compared to non-B, as well as in patients from W-Europe/N-America compared to those with other origins. A recent study reported a higher set-point virus load in patients with white ethnicity compared to black (mostly from sub-Sahara Africa), but no significant differences between infection with B and non-B subtypes [26]. Differences in the distribution of ethnicity and subtypes might explain this discrepancy.

Since only 30 of the 830 men we studied were infected through heterosexual contact, lack of power might have been a reason we found a non-significant difference in HIV RNA concentration at set-point between MSM and heterosexually infected patients. The SHCS reported a significant difference between MSM and heterosexually infected patients [8]. Also in contrast to the SHCS, our study and others found a higher HIV RNA concentration at set-point in female patients. The difference in HIV RNA concentration between men and women emerges only at lower CD4 cell counts [27], a factor that could explain these different results.

The HIV-1 RNA plasma concentration was measured with several assays. The distribution of the assays used has changed over the years, and we did not perform batch-wise re-testing of samples using only one assay. HIV-1 RNA concentrations measured within the dynamic range of the Versant HIV-1 RNA (bDNA) 3.0 assay are, on average, lower than those measured with the Cobas Amplicor assay (RT-PCR) [28]–[30]. The Amplicor HIV-1 Monitor assay (RT-PCR) [31] yields, on average, lower concentrations than the NASBA HIV-1 RNA QT assay, the only assay used in samples taken before 1996. This ranking was reflected in our analyses and might explain the more pronounced differences after adjusting for type of assay between seroconverters from 1984–1995 and from 2003–2007. In concordance with previous reports, the mean HIV-1 RNA concentration at set-point was slightly higher when measured using assays with a lower detection limit of 1000 or 400 copies/ml compared to ≤50 copies/ml [32], but adjustment for assay sensitivity did not appreciably change our results. The changing distribution of assays is thus unlikely to explain the increase in mean HIV-1 RNA concentration at viral set-point over time.

Techniques for measuring CD4 cell counts changed over time as well, leading to less test variability. Absolute CD4 cell counts were traditionally assessed using a dual-platform technique that has been gradually replaced by a single-platform technique introduced in the late 1990s. Others changes in flow cytometry techniques over time include changes in the gating strategy and sample preparation. There is some evidence to suggest that CD4 cell counts turn out lower when measured using the single-platform technique. Also, CD45-SSC gating, more frequently used in later calendar years, may yield higher CD4 cell counts than CD45-CD14 gating, more frequently used in earlier calendar years [33]. For our study, these two potential biases may outweigh each other.

In samples obtained before 1996, quantitation followed storage at −80°C. No significant effect of freezing, storage, and thawing on HIV RNA recovery using the Amplicor HIV-1 Monitor and NASBA HIV-1 RNA QT assay has been reported [31], [34], [35]. Moreover, we observed an increase in mean HIV-1 RNA concentration at set-point between periods 1996–2002 and 2003–2007.

The awareness level of physicians and patients to symptoms of acute HIV-1 infection has improved in recent years and could have influenced our findings. Set-point HIV-1 RNA concentration has been shown to increase with the number of symptoms after recent HIV-1 infection [36], [37]. In times when patients without symptoms were overlooked, the mean HIV-1 RNA concentration at set-point was most likely overestimated. However, as data on symptoms were not collected, we could not investigate this further.

The increasing number of patients included in our study over time positively correlated with increasing set-point viral load levels. The contribution of resulting selection of higher viral load at set-point in to facilitating the spread of HIV is a cause for alarm. However, other reasons for the higher number of study patients in more recent years include more sexual risk behaviour [38], [39], more HIV-negative subjects who repeatedly test for sexually transmitted infections (STIs) [39]–[41], and a higher incidence of STIs [42]. Also, the start in 2003 of a randomized study of patients with primary HIV-1 infection has raised physician awareness. Therefore, the higher number of new infections in recent years included in our study cannot be ascribed solely to a higher set-point viral load in those years. As for the other possibility, an ongoing phylogenetic study has found no indications that newly imported HIV-1 strains are responsible for the changes in set-point HIV RNA levels.

Some evidence suggests that HIV-1 RNA concentration at viral set-point is an adaptive trait and may change under the influence of selection [6]. Transmission of HIV-1 is thought to happen most efficiently very early, during primary infection, and late, during the symptomatic phase [4], [43]. During the asymptomatic phase after primary infection, the probability of transmission may be smaller, but given the long duration of this phase, its contribution to the number of newly infected patients is probably substantial. Successful antiretroviral therapy, usually started some years after primary infection, suppresses HIV-1 RNA plasma concentration to levels at which the probability of transmission is considered minimal. Thus the period of infectiousness may largely be confined to the very early phase of infection, in which viruses that reproduce at a high level are selected. Such viruses could establish a high HIV-1 RNA concentration at set-point in a new host [44]. For this hypothesis to hold, set-point viral load between transmitter and recipient needs to be correlated. This correlation will be investigated in a future study, using data from transmission networks.

A significant increase in HIV-1 replication fitness over time has been described for the Amsterdam epidemic [15]. Follow-up molecular analysis should reveal which changes in the viral components are responsible for this increased replication capacity. An increase in HIV RNA concentration at set-point may be also the result of adaptation of HIV to particular HLA molecules in the population. Kawashima *et al.* show that HLA molecules associated in 1983 with slow disease progression did not protect against disease progression in Japanese patients infected between 1997–2008 [45].

In conclusion, we found an increase in the HIV-1 RNA plasma concentration measured at viral set-point and a decrease in CD4 cell count in non-treated patients with a confirmed HIV-1 seroconversion during the last decade of the HIV epidemic in the Netherlands. The higher HIV-1 RNA concentration could not be attributed to changes in subtype or assay used, but coincides with a higher proportion of treated HIV-1-infected patients. The implications of an increased HIV-1 RNA concentration at viral set-point on disease progression and on transmission dynamics require further study.

## Methods

### Patient selection

The ATHENA observational cohort [46] includes anonomyzed data from all HIV- infected patients living in the Netherlands who receive care in one of the 24 HIV treatment centres. Initially, data were collected only from patients who had started combination antiretroviral therapy (cART). Data collection was extended in 2002 to include all HIV-infected patients, cART treated or untreated alike, who had been followed since 1996 in any of the centres. ATHENA patients are informed by their treating physician of an opt-out procedure. Ethical approval is not obtained, as data collection is part of HIV care. For our study, we also included data obtained from homosexual men (MSM) participating in the Amsterdam Cohort Studies (ACS) [47]. Informed written consent is obtained from all ACS participants, and the ACS has been approved by the Medical Ethical Committee of the Academic Medical Centre.

We selected patients with a maximum interval of one year between the last negative and first positive HIV-1 antibody test. The day of seroconversion was estimated as the midpoint between the last seronegative and the first seropositive test. Patients with negative or indeterminate Western Blot results in the presence of HIV-1 p24 anitigen or RNA were also selected, in which case the day of seroconversion was set one month prior to the date of the first positive antibody test. All patients in our study were at least 16 years old at the estimated day of seroconversion, had been sampled at least once for plasma HIV-1 RNA concentration between 9 and 27 months after seroconversion, and were antiretroviral therapy-naive. All patients who seroconverted before 1996 were participants of the ACS.

### Measurements

First, we used the HIV-1 RNA concentration and CD4 cell count measured in peripheral blood sampled earliest in the 9–27 months after seroconversion as the measurements at viral set-point. In addition, we selected results of the HIV-1 RNA and CD4 cell count measurements taken between 9 and 15 months (closest to 12), between 15 and 21 months (closest to 18), and between 21 and 27 months (closest to 24). Any measurements taken after the start of antiretroviral therapy were not used in any analysis. Assays were classified according to the amplification technique: nucleic acid sequence-based amplification (NASBA), reverse transcriptase-polymerase chain reaction (RT-PCR), and branched DNA signal amplification (bDNA). Assays using the NASBA technique were NASBA HIV-1 RNA QT, NucliSens HIV-1 RNA QT, and NucliSens EasyQ (bioMérieux, Boxtel, The Netherlands). Assays using RT-PCR techniques were Amplicor HIV-1 Monitor, Cobas Amplicor, Cobas TaqMan HIV-1 (Roche Diagnostics, Pleasanton, CA, USA), LCx HIV RNA quantitative, and m2000rt HIV RNA (Abbott, Abbott Park, IL, USA). The only assay using the bDNA technique was Versant HIV-1 RNA version 3.0 (Siemens, Deerfield, IL, USA). Classification by amplification technique is very similar to classification by manufacturer. The RT-PCR assays made by Roche and Abbott were grouped together. Since the Abbott assay was used in only 59 patients who all seroconverted in 2006 and 2007 these assays could not be analysed separately. Finally, HIV-1 RNA assays were also classified as standard (having a lower detection limit of 1000 or 400 copies/ml) and sensitive (having a lower detection limit ≤50 copies/ml).

### Statistical analysis

Parametric survival regression models with a normal error distribution were used to model changes in plasma HIV-1 RNA concentration at set-point. When below the limit of detection, the value was regarded as interval-censored between 1 copy/ml and the lower detection limit. Values above the upper detection limit were right-censored at the upper detection limit. CD4 cell count at viral set-point was modelled using linear regression models. CD4 cell counts were cube root-transformed to apply better to model assumptions. Estimated calendar year of seroconversion was modelled using 3 categories: <1996 (pre-cART), 1996–2002 and ≥2003. The post-cART cut-off was chosen so that each time period was sufficiently wide and included a sufficient number of patients. Estimated date of seroconversion was continuously modelled using restricted cubic splines with knots at the 5^th^, 50^th^ and 95^th^ percentiles. This allowed us to model date of seroconversion in a flexible manner and to avoid having to report results over year categories with few patients. Splines with 5 knots were also used but led to similar estimates. Potential confounders for analysis of HIV-1 RNA concentration at set-point included gender, region of origin (W-Europe/N-America, other, and unknown), age at seroconversion, HIV-1 subtype (B, non-B, and unknown), transmission of drug-resistant virus (at least one mutation, none, and unknown) [48], interval between measurement and seroconversion, transmission risk group (men having sex with men [MSM], heterosexual, intravenous drug use [IDU], other, and unknown), co-infection with HBV of HCV (positive, negative, or unknown), sensitivity of assay (lower detection limit ≥400 and <400 copies/ml), and technique of the quantitative HIV-1 RNA assay used (NASBA, RT-PCR, and bDNA). Active HBV co-infection was defined as a positive HBsAg test. Chronic HCV co-infection was defined as a positive HCV RNA test or, if not available, a positive HCV antibody test. We used the first available test result up to 2 years after the estimated HIV-1 seroconversion date. Variables were retained in adjusted models if their overall p-value was <0.20.

To obtain results in a population as homogeneous as possible, we also ran models including only data from MSM from W-Europe/N-America. Patients with a proven HIV-1 non-B infection were excluded. If the HIV-1 subtype was not determined, patients were included. In addition, we performed sensitivity analyses on patients with a confirmed subtype B infection. A final sensitivity analysis was performed on patients with a maximum seroconversion interval of 6 months, using the first HIV-1 RNA measurement after seroconversion as dependent variable. This analysis was performed in order to study the potential bias that results from censoring patients with an early start of antiretroviral therapy. Models were adjusted for time between the estimated day of seroconversion and the measurement using restricted cubic splines.

Analyses were performed using SAS version 9.1 (SAS Institute, Cary, North Carolina, USA).
